# First Report of Bronchial Oestriasis in a Domestic Dog

**DOI:** 10.1002/vms3.70583

**Published:** 2025-08-26

**Authors:** Filippo Maria Dini, Fabiano Raponi, Flavio Vallone, Roberta Galuppi, Fabio Macchioni

**Affiliations:** ^1^ Department of Veterinary Medical Sciences University of Bologna Ozzano Emilia Bologna Italy; ^2^ Endovet Italia Ancona Italy; ^3^ Department of Veterinary Sciences University of Pisa Pisa Italy

**Keywords:** bronchoscopy, dog, first instar larva, myiasis, *Oestrus ovis*

## Abstract

This study reports the first case of bronchial myiasis in a dog caused by *Oestrus ovis*, a parasite known to infest sheep, and occasionally other mammals. A 9‐year‐old dog, from a rural area near Ancona (Italy), exhibited persistent coughing despite antibiotic and anti‐inflammatory treatments. Bronchoscopy revealed a live first‐instar larva of *O. ovis*, confirmed through morphological and molecular analyses. Larval removal and treatment with isoxazolines resulted in a rapid resolution of symptoms. This case highlights the critical importance of including atypical parasitic infestations in the differential diagnosis of respiratory symptoms in dogs. It underscores the importance of heightened vigilance, particularly in areas with sheep flocks, to prevent and manage such infestation effectively.

## Graphical abstract

Oestrus ovis bronchial myasis is described in a dog for the first time.

Bronchoscopy proved essential for larval visualization and removal.

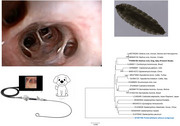

## 1 Introduction

*Oestrus ovis* Linnaeus, 1758, commonly known as the ‘sheep nasal bot fly’, is a cosmopolitan, zoonotic parasite belonging to the family Oestridae within the order Diptera. It is the primary causative agent of nasal myiasis, a pathological condition that primarily affects sheep and goats, but can also occasionally impact other mammals, including humans. The life cycle of *O. ovis* is direct and does not involve intermediate hosts. Adult female flies deposit larvae directly into the nostrils of their hosts through ‘larviposition’. Once inside the nasal passages, the larvae develop and migrate into the paranasal sinuses, causing irritation, inflammation, nasal discharge, and subsequent respiratory issues. After undergoing three developmental stages within the host, the mature larvae are expelled through sneezing or coughing. Once expelled, they form puparia in the soil within approximately 24 h, where they emerge as adult flies, completing the life cycle. This parasite is closely associated with sheep farming, with outbreaks typically occurring during hot and dry periods when the adult fly is most active (Gracia et al. 2019). *O. ovis* significantly threatens the sheep industry, reducing wool and meat production due to the stress and tissue damage caused by heavy infestations (Scala et al. 2001). Moreover, these infestations can compromise animal health, increasing the likelihood of secondary infections. In Italy, the parasite is widely distributed throughout the country. However, breeders often underestimate it, and updated data are lacking. Despite this, investigations have confirmed its presence in all regions where extensive sheep farming is practiced. Reported prevalence rates include 38% in Puglia (Nardi 1955), 89% in Abruzzo (Mantovani et al. 1964), 51% in Emilia‐Romagna (Pietrobelli and Capelli 1988), 55.8% in Sicily (Caracappa et al. 2000) and 91% in Sardinia (Scala et al. 2001).

While *O. ovis* involves sheep and goats in its life cycle, it can also affect other animal species, including humans, posing significant concerns for both veterinary and public health. In humans, accidental infestations have been well‐documented worldwide, with various localisations reported, including the pharynx (Hazratian et al. 2017), nose (Brini et al. 2019), ears (Al‐Dabagh et al. 1980), stomach (Yılmaz et al. 1999) and eyes (Tamponi et al. 2022).

Accidental oestriasis is occasionally reported in companion animals, such as cats (Webb and Grillo 2010) and relatively more frequently in domestic dogs. In dogs, *O. ovis* nasal myiasis has been sporadically documented worldwide, with symptoms including severe discomfort, nasal discharge, sneezing, and, in severe cases, secondary infections due to the larvae's presence (Zanzani et al. 2016). This paper presents, for the first time, a case of canine myiasis due to *O. ovis* with bronchial localisation.

## 2 Case Presentation

A 9‐year‐old mixed‐breed male dog from a rural area near Ancona, Italy, was referred to a veterinary clinic due to a persistent cough that had lasted for several days. An initial clinical examination and basic laboratory tests conducted did not reveal any significant abnormalities. The clinical examination showed no pathological findings, with body temperature within normal limits. Resting respiratory rate, recorded at home, was within normal parameters, whereas in the clinic, a paraphysiological increase was observed, likely due to stress induced by the visit.

Suspecting an inflammatory or infectious cause, the dog was empirically treated with amoxicillin‐clavulanate at 20 mg/kg BID and meloxicam at 0.1 mg/kg SID. However, the condition did not improve despite this intervention. Due to the lack of response to treatment and the ongoing respiratory symptoms, the dog was referred for further diagnostic evaluation, including an endoscopic examination to rule out the presence of a bronchial foreign body. During bronchoscopy, a live larva was observed actively moving within the bronchial tree in LB2V1, one of the first branches of the left main bronchus (see Supporting Information S1). The only recovered larva extracted during the endoscopic procedure was initially observed microscopically alive in a drop of saline solution, then fixed in ethanol at 70°C for further microscopic and molecular analysis. The larva was initially measured and then dissected under a stereoscope. The anterior and posterior ends were mounted on a slide in lactophenol, while a small segment was preserved for DNA extraction, which was performed using PureLink Genomic DNA Mini Kit (Invitrogen, Thermo Fisher), following the manufacturer's protocol. The amplification of the COI gene was carried out according to Tuccia et al. (2016). Amplifications were conducted using a T‐personal thermal cycler (Biometra, Göttingen, Germany). The PCR products were electrophoresed on a 1% agarose gel stained with SYBR Safe DNA Gel Stain (Thermo Fisher Scientific, Carlsbad, CA, USA) in 0.5× TBE buffer. For sequencing, the amplicons were excised, purified using the NucleoSpin Gel and PCR Cleanup kit (Mackerey‐Nagel, Düren, Germany), and sequenced using an ABI 3730 DNA analyser (StarSEQ, Mainz, Germany). The trace files were assembled with Contig Express (VectorNTI Advance 11 software, Invitrogen, Carlsbad, CA, USA), and the consensus sequence was compared with published data using BLAST tools (https://blast.ncbi.nlm.nih.gov/Blast.cgi) (accessed on 10‐08‐2024). Sequence alignment was performed using BioEdit 7.2.5 (Hall 1999), and the phylogenetic tree was constructed using the Neighbour‐Joining method with MEGA7 software (Kumar et al. 2016). The accession numbers of the sequences included in the analyses are provided within the phylogenetic tree. This comprehensive approach ensured a thorough characterization of the specimen.

## 3 Results and Discussion

**FIGURE 1:**
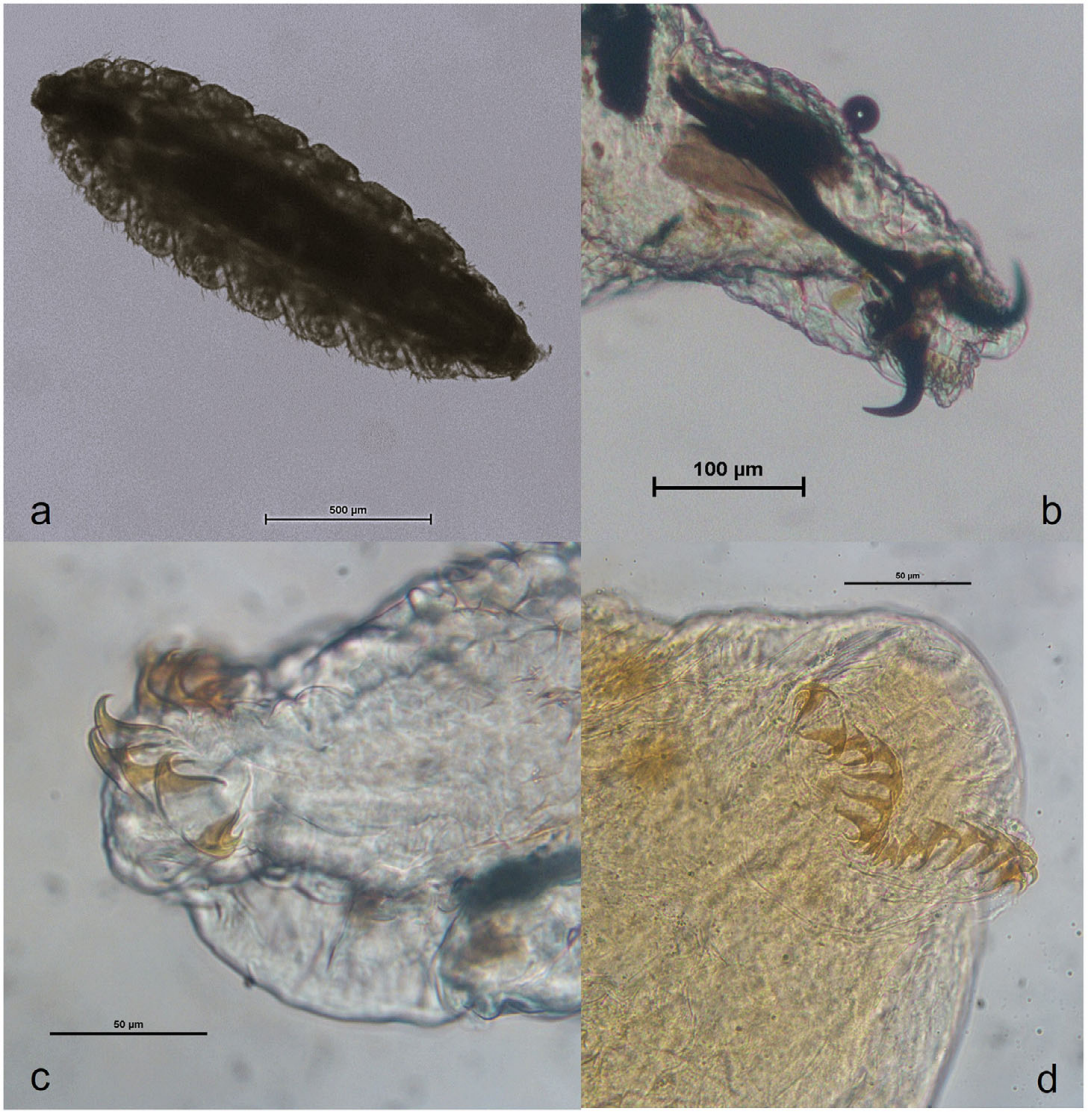
*Oestrus ovis* first instar collected from the dog after bronchoscopy: (a) total body (× 100); (b) anterior end with cephaloskeleton (× 200); (c) detail of the posterior end, partially lateral view (× 400); (d) detail of the posterior end after full clarification, with nine hooks on each side: ventral view (× 400).

At microscopic observation, a first‐instar dipteran larva was recognizable. The larva appeared spindle‐shaped and measured 1.734 mm in length and 0.555 mm in width. The body was divided into 12 distinct segments. Laterally, the posterior part of each metamere was provided with bunches of small bristles. The cephalic end, corresponding to the first metamere, also called pseudocephalon, bore two strong, black, sclerotic, curved buccal hooks in the shape of a bull's horns, as part of the cephaloskeleton. The caudal segment was elongated, bearing nine small cat's claw hooks, curved ventrally on either side of the midline (Figure 1). The larva's morphological characteristics were consistent with first instar of *O. ovis*.

**FIGURE 2:**
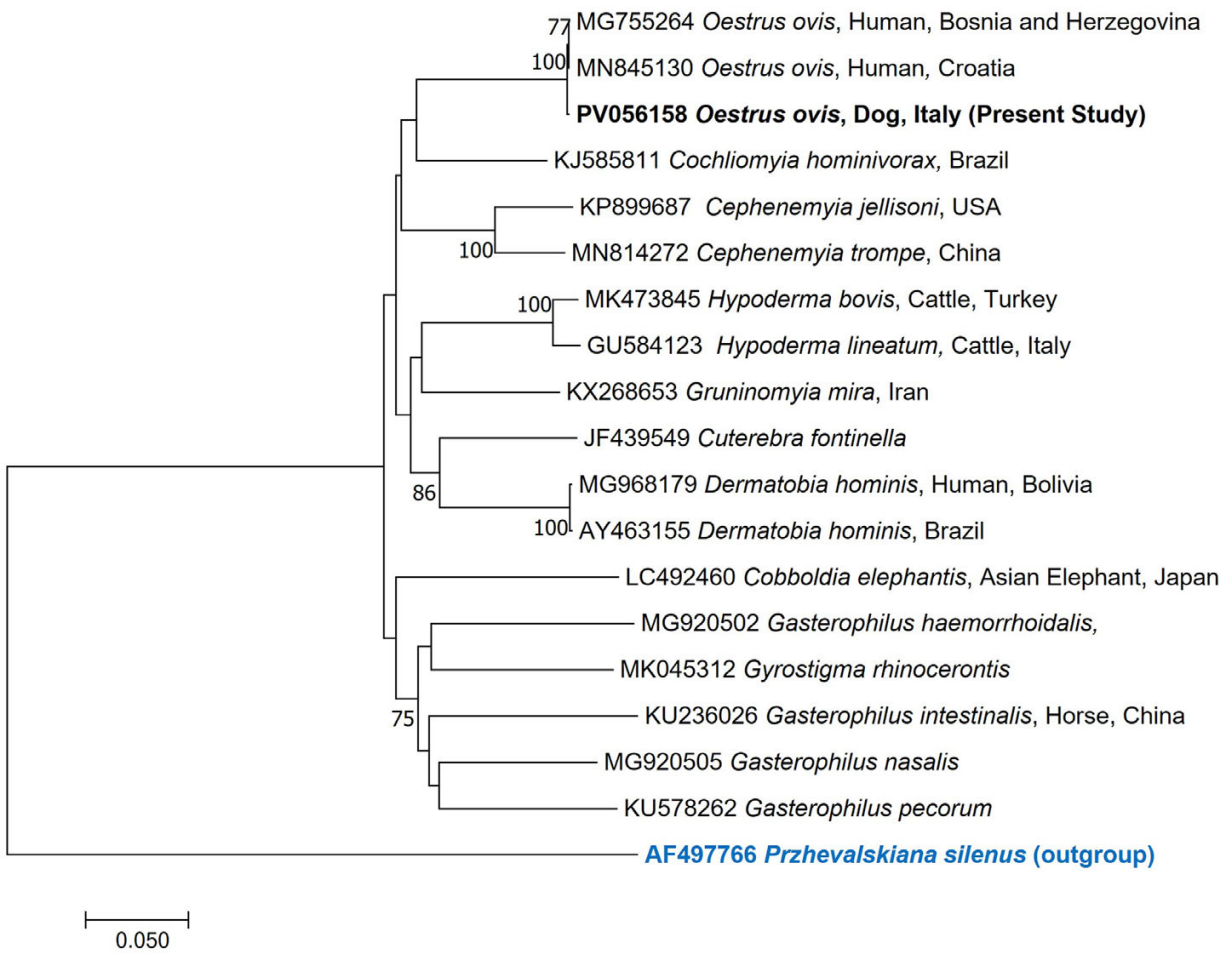
Neighbour‐Joining phylogenetic tree based on the p‐distance model. The tree is drawn to scale, with branch lengths measured in the number of substitutions per site. The analysis involved 19 nucleotide sequences. There were 644 positions in the final dataset.

Sequencing of the COI amplicon yielded 99.8%–100% similarity to *O. ovis* sequences available on GenBank, as determined by BLAST search. In the phylogenetic analysis, our sequence clustered with *O. ovis* sequences from Europe, with high bootstrap values strongly supporting the separation of our sequence from those belonging to different families of flies (Figure 2).

Following the larva extraction, the dog experienced a remission of symptoms. However, it was treated with an oral dose of isoxazoline medication (Lotilaner, Credelio), in case additional larvae were not visible during the endoscopy. Remarkably, within 36 h of the initial dose, the dog's coughing resolved completely. Follow‐up evaluations over the subsequent weeks showed no recurrence of clinical signs, indicating the successful eradication of the infestation.

Although *O. ovis* predominantly targets small ruminants, aberrant infestations can occur in other species under specific environmental conditions (Luján et al. 1998). In dogs, occurrence of nasal myiasis caused by *O. ovis* are typically reported in regions where sheep farming is prevalent, and increasing reports potentially reflecting shifts in the parasite's geographic distribution and environmental conditions. One of the earliest documented cases when Lucientes et al. (1997) reported a case in the Canary Islands involving a dog that expelled a third‐instar larva through sneezing. This was followed by Luján et al. (1998), who documented the recovery of *O. ovis* third instar larvae from a dog's nasal cavity during a necropsy in Spain. Heath and Johnston (2001) reported a case in New Zealand, where a first‐instar larva was recovered from a dog's nasal cavity using endoscopy. Later, McGarry et al. (2012) described the first report of *O. ovis* infestation in a dog in the UK. The case involved a farm dog in the Cotswolds that expelled a third‐instar larva following violent sneezing episodes. In northern Italy, Zanzani et al. (2016) documented a case involving a dog presenting with frequent and severe sneezing, where a larva of *O. ovis* was detected during rhinoscopy. Vergles Rataj et al. (2021) reported the first case of *O. ovis* nasal infestation in a dog in Slovenia; this urban dog was presented with acute sneezing and bilateral nasal discharge, and rhinoscopy revealed the presence of first‐instar larvae. Most recently, Azoulay et al. (2023) in France described a case involving a Cane Corso dog with chronic epistaxis, reverse sneezing, and purulent nasal discharge. The diagnosis was achieved using CT scans and a frontal osteotomy, and larval removal combined with oral isoxazoline therapy led to a complete resolution of clinical signs. These cases highlight the growing geographic spread and clinical impact of *O. ovis* infestations in dogs.

In the present case, a bronchial localization of first‐instar *O. ovis* larvae was observed for the first time. In this instance, the dog's environment featured regular transhumance, with sheep flocks frequently passing through or temporarily residing near the property, particularly one that had recently occupied adjacent land, which likely facilitated the opportunistic infestation. The clinical presentation in this case was non‐specific, with coughing as the primary symptom. These signs are easily mistaken for more common respiratory conditions, potentially delaying accurate diagnosis and treatment. Bronchoscopy proved essential for visualizing and identifying the larva, highlighting the importance of advanced diagnostic tools in atypical cases. During the endoscopic examination, only one specimen was observed; however, the presence of additional larvae could not be excluded, prompting the administration of treatment. Clinical recovery was achieved 36 h post‐treatment with isoxazolines. These compounds are widely recognized for their broad‐spectrum efficacy against ectoparasites and have been used off‐label for treating dog rhinomyiasis (Azoulay et al. 2023). Isoxazolines, including lotilaner, exert both insecticidal and acaricidal activities by acting on specific GABA/glutamate receptors, inhibiting chloride ion channels, which leads to hyperexcitation and death of the parasite (Ozoe et al. 2010). While primarily indicated for flea and tick control, the efficacy of lotilaner has also been documented against other ectoparasites, such as dipteran larvae (do Vale et al. 2023), making it a valuable option for managing such infestations.

## 4 Conclusion

This report emphasizes the importance of heightened awareness in diagnosing rare parasitic infestations in domestic animals, especially in areas where cross‐species transmission is common. Early detection and timely therapeutic intervention are crucial for successful outcomes. Regular monitoring and preventive measures, such as reducing exposure to potential infestation sources and considering prophylactic antiparasitic treatments, are recommended for dogs living in or near livestock environments. Veterinarians practicing in rural areas should maintain a high level of suspicion for atypical parasitic infections in dogs presenting with unexplained respiratory symptoms, particularly when there is known contact with livestock. Isoxazoline compounds should be considered both as a treatment and as a preventive option for a range of ectoparasitic infestations. Finally, further studies are needed to develop standardized management protocols for handling such rare cases of myiasis in canine patients.

## Author Contributions

**Filippo Maria Dini**: investigation, methodology, writing – original draft preparation, writing – review and editing. **Fabiano Raponi**: resources, investigation, writing – original draft preparation, writing – review and editing. **Flavio Vallone**: investigation, writing – review and editing. **Roberta Galuppi**: investigation, visualisation, writing – original draft preparation, writing – review and editing. **Fabio Macchioni**: writing – original draft preparation, supervision, writing – review and editing.

## Ethics Statement

The sample was collected during clinical activities performed in accordance with relevant guidelines and regulations, no specific permission was required to perform the sampling.

## Consent

The authors have nothing to report.

## Conflicts of Interest

The authors declare no conflicts of interest.

## Supporting information




**Supporting File 1**: vms370583‐sup‐0001‐VideoS1.mp4

## Data Availability

All data supporting the present study are shown in the manuscript.
